# Tuberculosis, a Disease of Children

**Published:** 1917-10

**Authors:** A. Clifton Moor

**Affiliations:** Tallahassee, Fla.


### Journal-Record of Medicine

Successor to Atlanta Medical and Surgical Journal, Established 1855
and Southern Medical Record, Established 1870

**OWNED BY THE ATLANTA MEDICAL JOURNAL COMPANY**

PUBLISHED MONTHLY

*Official Organ Fulton County Medical Society, State Examing Board, Atlanta, Birmingham and Atlantic Railroad Surgeon's Association, Chattahoochee Valley Medical and Surgical Association, Etc.*

A. W. STIRLING, M. D., C. M., D. P. H.; J. S. HURT, B. Ph., M.D.
GEO. M. NILES, M. D., W. J. LOVE, M. D., (Ala.); Associate Editors

E. W. ALLEN, Business Manager

**COLLABORATORS**

W. F. WESTMORELAND, M. D., General Surgery
F. W. McRAE, M. D., Abdominal Surgery
ALLEN H. BUNCE, Medical Societies
E. B. BLOCK, M. D., Diseases of the Nervous System
MICHAEL HOKE, M. D., Orthopedic Surgery
CYRUS W. STRICKLER, M. D., Legal Medicine and Medical Legislation
E. C. DAVIS, A. B., M. D., Obstetrics
E. G. JONES, A. B., M. D., Gynecology
ALLEN H. BUNCE, M. D., Pathology and Bacteriology
R. T. DORSEY, JR., B. S., M. D., Medicine
L. M. GAINES, A. B., M. D., Internal Medicine
GEO. C. MIZELL, M. D., Diseases of the Stomach and Intestines
L. B. CLARKE, M. D., Pediatrics
EDGAR PAULLIN, M. D., Opsonic Medicine
THEODORE TOEPEL, M. D., Mechano Therapy
E. G. BALLENGER, M. D., Diseases of the Genito-Urinary Organs

VOL. LXIV. ATLANTA, GA, OCT., 1917. NO. 7

### TUBERCULOSIS, A DISEASE OF CHILDREN.

**By A. Clifton Moor, M.D., Tallahassee, Fla.**

The importance of tuberculosis in early life is on of the subjects which has received especial consideration in latter years. That it makes up no small part of the mortality during childhood is shown by the autopsy findings in practically all medical centers. Still reports tuberculosis lesion in 269 out of 969 consecutive autopsies in the Ormond Street Children's Hospital, and states that in 223 of these tuberculosis was the immediate cause of death. While in New York, in a series of 1320 autopsies in children, all under five years of age, 13.5 per cent. showed more or less generalized tuberculosis disease. The mortality among private patients is certainly much less than this, but there is no doubt that many deaths from the acute type, which characterizes tuberculosis in nurslings, are attributed to other causes.

However, it is tuberculosis infection rather than tuberculous disease which adds greatly to the importance of the first decade of life. Von Behring originally advanced the view that all primary tuberculous infection occurred before puberty, and the newer tuberculin tests have enabled us to ascertain, with a reasonable degree of accuracy, the number of latent cases and to recognize some of the obscure manifestations of the disease.

Assuming that a positive cutaneous test is evidence of hypersensibility brought about by the presence of tubercle bacilli in thebody tissues, and comparing the results of these tests with autopsy reports and similar tests in adults, we are impressed with the fact that the per cent. of positive reactions at puberty is practically the same as at any later period of life.

The largest series of reports come from Europe, where the number of positive reaction is considerably higher than in this country. But the opinion of American investigators bears out the conclusion, that, making due allowance for difference in home hygiene and better living conditions, there is the same relative proportion of infected children and tuberculosis adults here as in Europe.

Dr. G. R. Pisek, of New York, gives as his opinion that 60 per cent. of all infections occur before the sixth year, and 70 per cent. before the fourteenth year. Dr. Alfred Hess, of New York, thinks that over 70 per cent. of the infections occur before the third year; and Prof. Hamburger, of Vienna, states that 51 per cent. are infected during the first year of life, and 94 per cent. by the end of the fourteenth, while results of autopsies in supposedly non-tuberculosis adults show that from

59 to 92 per cent. have evidence of tuberculous disease somewhere in the body.

For many years tuberculosis was believed to be hereditary. This view was held by the layman and profession alike; and, while we now know that only occasionally is a child actually born tuberculous, the clinical facts are the same today as during the last generation—that is, that tuberculosis is a family disease, and the large majority of intelligent tuberculous patients can give a history of definite exposure to other cases in the household during their own childhood.

The near relation of children to tuberculous cases in the family is necessarily intimate; and it naturally follows that the greatest factor in the infection of children is the presence of active disease in either parents or grandparents. If to this be added a crowded and poorly ventilated home and food of insufficient quality or quantity, there is practically no chance for the child to escape.

Prof. John Howland, of Baltimore, Drs. Holt, Biggs and Chapin, of New York, and Lucas, of San Francisco, believe that 90 per cent. of the infections in childhood are the result of close personal contact with the tuberculous individuals; and this seems to represent the majority opinion of the profesion today.

The effect of good home conditions on the percentage of childhood infections is shown by Cattermole in a small series of tests among private patients in Colorado, where, with over half of the patients suffering from active tuberculous disease, only 38 per cent. of the exposed children and less than 1 per cent. of the unexposed gave positive tuberculin reactions.

Fishburg gives us the reverse of this picture from the tenement district of New York, where he found that 67 per cent. of the children living with tuberculous parents and over 52 per cent. of those with non-tuberculous parents gave positive reactions. This would indicate not only a widespread prevalence of virulent bacilli in the tenement homes, but also a low resistance of tenement children; and the conclusion is justifiable that few of them reach puberty without infection.

In this connection it might be of interest to quote the figures of Knopf, who states that 82 per cent. of the cases of tuberculosis in Berlin occur in families occupying two rooms or less and only 6 per cent. in families occupying four rooms or more.

Aside from the part undernourishment plays in increasing the general susceptibility to disease, food is a direct etiological factor, but just how much importance bovine tuberculosis may have in the production of the disease is still a mooted question. The British and German Commission found the bovine type in 13 per cent. of the cases in children, and Park and Krumwilde, from data obtained in fifteen hundred of their own and collected cases, conclude that over 10 per cent. of the tuberculous disease in children under five is of bovine origin.

The report of the United States Bureau of Animal Industry, that between 20 and 30 per cent. of the dairy cattle of this country are tuberculous, would indicate that there is opportunity for a widespread infection from this source. That such does not occur must be attributed to the fact that it is only when there is disease of the udder or active ulceration of the intestinal or genit-urinary tract that the purity of the milk is endangered.

The bovine bacillus seldom remains latent in the human and adults seem practically immune to it. In the child the most common clinical manifestations of bovine infection are the cases of so-called lymphadenitis and diseases of the bones, joints and abdomen.

The location of the earliest lesions of tuberculosis when such lesions can be demonstrated, would naturally depend very largely upon the route of infection, that is, whether the disease is acquired through the respiratory or alimentary tract. Wollenstein and Bartlett report that in 178 children under five years of age, in which the initial lesion could be ascertained with reasonable certainty, 135 showed the earliest signs of disease in the thorax. All statistics go to prove that early and marked envolvement of the tracheo-bronchial and, to a less extent, the mesenteric lymph glands, is the rule. And here, no doubt, is the site of the latent disease, which only awaits a more favorable local or constitutional condition to again become active.

In a brief discussion of this kind it is manifestly impossible to more than outline the more common types of the disease peculiar to childhood, and to call attention to the most common symptom and diagnostic points.

The symptomatology and course varies widely. In older children the symptoms differ little from those seen in the adult; but in nurslings the disease shows a marked tendency to early generalization and progresses rapidly to a fatal termination. The prognosis during the first year is absolutely bad, and during the second year a large per cent. of the cases end with meningitis.

The clinical types to be especially noted are generalized tuberculosis, and disease of the thoracic glands, of the lungs and of the abdomen.

In the diagnosis of tuberculosis of any form the cutaneous test is of value. In infancy a positive reaction undoubtedly means active disease; for latent conditions do not exist before the third year. In other children a positive reaction aids in confirming a suspicion of tuberculosis when there are definite clinical symptoms and physical signs; and repeatedly negative reactions will serve to eliminate tuberculous disease from further consideration in the diagnosis.

Another symptom of value, and one which is frequently overlooked, is the presence, in a considerable per cent. of cases, of certain characteristic skin lesions. The tuberculides are not difficult of recognition and are often the earliest definite evidence of tuberculosis. The more common clinical types are lichen scrofulorosum, scrofuloderma, erythema induratum and the papulo-neucrotic and papulo-squamous forms. These lesions may be isolated and few in number, but their morphology and sites of predeliction should be kept continually in mind in all routine examinations.

A history of exposure to open tuberculosis in other members of the family is of the greatest diagnostic importance, and particularly if the child is under five years of age and the exposure has lasted over a considerable period of time.

General tuberculosis is often insidious in its onset, and in nurslings the symptoms are frequently those of a general wasting. As a rule a carefully kept temperature record will show the presence of elevated temperature, although this is seldom constant, or regular in its course. It should serve, however, to raise the suspicion of tuberculosis, because marasmus, as a result of chronic alimentary disease, runs a subnormal temperature.

Late in the disease there may be recognized more or less definite signs in the lungs or outspoken tuberculosis elsewhere. But frequently the development of meningitis is the first suspicious symptom.

It is in these cases that the tuberculin test and frequent search for the skin lesions may prove most helpful.

In other instances the clinical picture is more nearly that of an acute infection, with a sudden onset and a rapid rise of temperature. There may be the physical sign of a bronchitis, but the general appearance of a toxic pneumonia.

Tuberculous disease of the lung may be divided into two groups—first, chronic pulmonary tuberculosis, which differs little from the phthisis of adults, except that the disease spreads from the deep-seated bronchial or pulmonary glands and the earliest lesions are frequently in the base or near the root of the lung; and secondly, acute tuberculous bronchopneumonia, which is by far the most common and characteristic clinical type of tuberculosis in early life.

Two symptoms of pulmonary tuberculosis in the adult are of no diagnostic value in children, viz: sweating and hemoptysis. Children sweat easily from any cause, and rickets, debility from acute illness, or severe anemia, may be ample explanation of profuse night sweats.

Hemoptysis is a rare complication of tuberculosis in children. It is much more likely to occur as a complication of whooping cough or pulmonary congestion with advanced heart disease.

Cough is a more or less constant symptom, but is present in too many other conditions to be of much diagnostic value. Holt calls attention to the possibility of sputa examination even in very young children and states that bacilli were found in 80 per cent. of his cases. His method of collecting sputa is to incite cough by irritating the pharynx with a cotton or gauze swab to which the sputa will adhere.

In tuberculous broncho-pneumonia the symptoms and physical signs do not differ from a simple broncho-pneumonia, and delayed resolution is often the first thing which raises a suspicion of tuberculosis.

In the less acute cases paravertebral dulness and changes in the breath sounds in the inter-scapular region are suggestive of tuberculous disease. And if these be associated with elevated temperature, rales and a positive Von Pirquet we may feel reasonably sure of the diagnosis.

We have already noted that disease of the bronchial lymph glands is the most frequent manifestation of tuberculosis in children. This, however, is from the pathological view point. Clinically, such lesions may exist without symptoms or only be suspected when secondary infection occurs. When, however, there is pressure upon some bronchus or irritation of the vagus or phrenic nerve, cough and dyspnea are apt to develop. These constitute the most characteristic symptoms. The cough is dry, sometimes croupy, and often paroxysmal, resembling pertussis. The dyspnea is most marked on expiration and is frequently intermittent.

Enlargement of the superficial veins over the upper thorax may be noted and is especially significant if unilateral. Examination usually shows relative dullness on one or the other side of the sternum at the level of the third or fourth rib or in the inter-scapular space. The most constant sign is vertebral bronchophony at a lower level that is seen in health, extending in some cases as far down as the sixth thoracic vertebra (D'Espines' sign).

The course of disease of the thoracic glands is variable. Encapsulation or calcification may occur, or the glands may go on to caseation and ulcerate into a bronchus, setting up an acute process in the lungs, or again extention through the eral or local resistance and lights up the infection. Owing to lymph channels or blood vessels may set up an acute generalized tuberculosis. The disease focus may remain latent for an elar or local desistance and lights up the infection. Owing to the bronchial catarrh accompanying them, measles, whooping cough and influenza are the most frequent sources of trouble.

Abdominal tuberculosis, when the result of bovine infection, is usually primary, but it may be and often does occur as a secondary condition to thoracic disease. It may be a part of general miliary tuberculosis, in which case the onset is sudden and the symptoms are those of an acute general peritonitis. When, however, the peritonitis becomes infected from the mesenteric glands, the onset is more gradual and the course is distinctly chronic. There is always marked emaciation and usually a decided afternoon elevation of temperature. There may be free fluid in the peritoneal cavity, but the plastic type is perhaps the more common clinical variety. In this type there develops the "doughy" feeling to the whole abdomen and the characteristic swelling and inflammation at the umbilicus. The differential diagnosis depends upon the history of exposure, the course of the disease and the result of the tuberculin test.

The isolation of all cases of tuberculosis and the destruction of all inefective bacilli may be a "consummation devoutly to be wished for," but it is manifestly impossible.

If the first decade of life is the period of the greatest susceptibility to infection, and such seems to be undoubtedly the general belief, prophylaxis of tuberculosis in childhood becomes the most worthwhile proposition that confronts the medical profession. It simplifies the whole question of prevention, and concentrates our effort along more or less definite lines. As a matter of fact, in the general campaign against tuberculoosis the child is given only passing consideration.

The first requisite for the proper institution of preventive measures is a complete census of the tuberculous cases and the enforcement of a law requiring all new cases to be reported. In connecton with these reports there should be data as to the home conditions of the patients, number of room available for occupancy, number of indivduals in the family, and especially the number and ages of the children. This data should be arranged on a unit basis. With such an arrangement, one district nurse to each unit, could make the rounds often enough to keep in touch with the conditions of each family, and combine, to a certain extent, the duties of a visiting tuberculosis nurse and child welfare worker.

The cost to each unit would not exceed the average tax now paid for the control and punishment of petty criminals, and while we will admit the undesirable character of chicken thieves and ''crap-shooters,'' their total elimination is of small importance as compared with the prevention of even a part of our tuberculous mortality.

The points to be stressed are ample protection for all children under five, and prevention of the massive infection, which is sure to occur when there is active disease in either parent or some other member of the household. No mother with active disease should nurse her infant, and she and others of the household should be taught by precept and example all the detailed precautions necessary.

In a certain per cent. of the families where ignorance and poverty or a bedridden mother makes home conditions hopeless, there is nothing to be considered except the removal of the younger children to a ''preventorium,'' the establishment of which is one of the first problems that confronts us.

The second and less important requirement in preventing infection is the control of bovine tuberculosis. This can be done only by uniform state laws or, better still, by the Federal Government. At present the Federal law prohibits the interstate shipment of tick-infected cattle into tick-free territory, but places no restriction upon the movement of tuberculous cows; and each state has individual and widely differing regulations.

In addition to the measures to minimize the dangers of infection per se, there are others which will improve the general physical well-being and increase the tissue resistance against active disease. These measures are applicable especially during school age and demand, in addition to uniform school inspection, a school medical advisor. In fact, the physical fitness of the school child is of the greatest importance from every standpoint and should receive as much consideration as his mental training. Every child who is anemic, under weight, scrofulous or otherwise below par, should be given the advantage of open air schools, and should receive the especial care of the medical advisor.

In my own state there has been recently instituted work along this line. Beginning in 1914 with two nurses for the whole state, the number has been increased to thirteen, twelve of whom are assigned to definite districts. Previous to the institution of this plan there were practically no cases reported to the board of health. During the first year of the present campaign 1225 cases were seen by the visiting nurses and up till September of this year 2781 patients are or have been visited and instructed in the principles of prevention. The average time between visits is four weeks, and the nurses report that the great majority of the families visited are interested and really endeavor to follow instruction.—Pediatrics.

